# Optimizing Systems for Cas9 Expression in Toxoplasma gondii

**DOI:** 10.1128/mSphere.00386-19

**Published:** 2019-06-26

**Authors:** Benedikt M. Markus, George W. Bell, Hernan A. Lorenzi, Sebastian Lourido

**Affiliations:** a Whitehead Institute for Biomedical Research, Cambridge, Massachusetts, USA; b University of Freiburg, Faculty of Biology, Freiburg, Germany; c J. Craig Venter Institute, Rockville, Maryland, USA; d Department of Biology, Massachusetts Institute of Technology, Cambridge, Massachusetts, USA; University at Buffalo

**Keywords:** CRISPR, Cas9, *Toxoplasma gondii*, genome editing

## Abstract

CRISPR-Cas9 technologies have enabled genome engineering in an unprecedented array of species, accelerating biological studies in both model and nonmodel systems. However, Cas9 can be inherently toxic, which has limited its use in some organisms. We previously described the serendipitous discovery of a single guide RNA (sgRNA) that helped overcome Cas9 toxicity in the apicomplexan parasite Toxoplasma gondii, enabling the first genome-wide loss-of-function screens in any apicomplexan. Even in the presence of the buffering sgRNA, low-level Cas9 toxicity persists and results in frequent loss of Cas9 expression, which can affect the outcome of these screens. Similar Cas9-mediated toxicity has also been described in other organisms. We therefore sought to define the requirements for stable Cas9 expression, comparing different expression constructs and characterizing the role of the buffering sgRNA to understand the basis of Cas9 toxicity. We find that viral 2A peptides can substantially improve the selection and stability of Cas9 expression. We also demonstrate that the sgRNA has two functions: primarily facilitating integration of the Cas9-expression construct following initial genome targeting and secondarily improving long-term parasite fitness by alleviating Cas9 toxicity. We define a set of guidelines for the expression of Cas9 with improved stability and selection stringency, which are directly applicable to a variety of genetic approaches in diverse organisms. Our work also emphasizes the need for further characterizing the effects of Cas9 expression.

**IMPORTANCE** Toxoplasma gondii is an intracellular parasite that causes life-threatening disease in immunocompromised patients and affects the developing fetus when contracted during pregnancy. Closely related species cause malaria and severe diarrhea, thereby constituting leading causes for childhood mortality. Despite their importance to global health, this family of parasites has remained enigmatic. Given its remarkable experimental tractability, T. gondii has emerged as a model also for the study of related parasites. Genetic approaches are important tools for studying the biology of organisms, including T. gondii. As such, the recent developments of CRISPR-Cas9-based techniques for genome editing have vastly expanded our ability to study the biology of numerous species. In some organisms, however, CRISPR-Cas9 has been difficult to implement due to its inherent toxicity. Our research characterizes the basis of the observed toxicity, using T. gondii as a model, allowing us to develop approaches to aid the use of CRISPR-Cas9 in diverse species.

## Abstract (precis)

Toxoplasma gondii is an intracellular parasite that causes life-threatening disease in immunocompromised patients and affects the developing fetus when contracted during pregnancy. Closely related species cause malaria and severe diarrhea, thereby constituting leading causes for childhood mortality. Despite their importance to global health, this family of parasites has remained enigmatic. Given its remarkable experimental tractability, T. gondii has emerged as a model also for the study of related parasites. Genetic approaches are important tools for studying the biology of organisms, including T. gondii. As such, the recent developments of CRISPR-Cas9-based techniques for genome editing have vastly expanded our ability to study the biology of numerous species. In some organisms, however, CRISPR-Cas9 has been difficult to implement due to its inherent toxicity. Our research characterizes the basis of the observed toxicity, using T. gondii as a model, allowing us to develop approaches to aid the use of CRISPR-Cas9 in diverse species.

## INTRODUCTION

While CRISPR-Cas9 technologies have enabled genome editing in an unprecedented array of species, their use is often limited by the cytotoxic effects of heterologous Cas9 expression. CRISPR naturally occurs as an adaptive immune system in bacteria and archaea, where it degrades foreign, potentially harmful, DNA (1, 2). The type II CRISPR system from Streptococcus pyogenes was adapted as CRISPR-Cas9 technology for targeted genome engineering and has been applied in numerous organisms (3, 4). This CRISPR system can be reduced to two components: (i) the endonuclease Cas9 and (ii) the chimeric RNA, termed single guide RNA (sgRNA). Together, Cas9 and the sgRNA form a ribonucleoprotein complex that induces DNA double-strand breaks (DSBs) at target loci. A 20-nucleotide (nt) sequence at the 5′ end of the sgRNA, the protospacer, determines the specificity of the DNA target by Watson-Crick base-pairing. Changes in the protospacer sequence program Cas9 to target any DNA sequence of interest as long as it is followed by the trinucleotide NGG or NAG, which is referred to as the protospacer adjacent motif (PAM). The utility of using sgRNAs for site-specific nuclease activity, combined with the high efficiency of this technique, has proven transformative for gene manipulation in many organisms.

The protozoan parasite Toxoplasma gondii is an obligate intracellular pathogen of the phylum Apicomplexa, which comprises many other important pathogens of humans and livestock, including *Plasmodium* and *Cryptosporidium* spp. Despite their importance to global health, apicomplexan parasites have remained enigmatic. Only a few species have been studied, and less than half of their genes have been functionally annotated. Due to the ease of continuous cell culture and the availability of tools for forward and reverse genetics, T. gondii has become a model organism for the study of the apicomplexan phylum. Previously, transient expression of S. pyogenes Cas9 (Cas9) in T. gondii has enabled editing and disruption of genes with unparalleled ease and efficiency (5, 6). Motivated by the pioneering work in mammalian cell culture systems (7–9), we sought to use the CRISPR-Cas9 system to conduct genome-wide loss-of-function (LOF) screens in order to measure the fitness contribution of the many still-uncharacterized T. gondii genes. Generating a stable Cas9-expressing (Cas9^+^) strain was instrumental in achieving the efficiency of gene disruption needed for these screens. However, our initial attempts to constitutively express Cas9 failed, implying an inherent toxicity of the transgene in T. gondii that prevents the use of conventional approaches for Cas9 expression.

Cas9 toxicity has likewise been implicated in other species, where it has limited or even precluded the use of CRISPR-Cas9 for genome editing. In fact, heterologous Cas9 expression was shown to impair cell growth in most prokaryotes (10–12). Similar reports demonstrated the impact of Cas9 expression on the growth of eukaryotes, such as Saccharomyces cerevisiae, Trypanosoma cruzi, and Trichomonas vaginalis (13–15). While Cas9 toxicity appears to depend on its catalytic activity in some species (16, 17), in others, toxicity is nuclease independent (14, 18, 19), suggesting that the causes of Cas9 toxicity are manifold and difficult to disentangle. As with any transgene, high-level expression of Cas9 or nuclease-deficient Cas9 (dCas9) can result in general proteotoxic stress (19). Recent studies that investigated the DNA-binding kinetics of dCas9 demonstrated that the protein can bind PAM sequences nonspecifically—especially in the absence of an sgRNA—which may explain the deleterious effects of both Cas9 and dCas9 (20–22).

We previously described that coexpression of a specific sgRNA (sgRNA #1) was able to overcome Cas9 toxicity in T. gondii. It was only when we coexpressed this sgRNA that we were able to isolate stable Cas9^+^ cells. However, the mechanism behind the apparent relief of Cas9 toxicity by the sgRNA #1 remained unknown. Since publication of our previous study (23), we have observed the frequent spontaneous loss of Cas9 expression from clonal T. gondii strains. The subsequent rapid takeover of Cas9-negative (Cas9^–^) cells compromises the efficiency of gene disruption needed for large-scale screens. The inferred fitness defect of Cas9^+^ cells further corroborates the deleterious effect of Cas9 and indicates that Cas9 toxicity is not fully relieved by the sgRNA #1.

In this study, we develop a system for Cas9 expression with improved selection and transgene stability. The use of viral 2A peptides that allow for more stable transgene expression enabled us to study the requirements of the coexpressed sgRNA for generating Cas9^+^ cells. We find that a genome-targeting sgRNA can vastly increase the rate of successful expression construct integration and define the site of integration via nonhomologous end joining (NHEJ). This expression system further allowed us to generate Cas9^+^ cells in the absence of an sgRNA. We determined that these cells displayed a fitness defect compared to cells expressing the sgRNA #1, suggesting a role for the sgRNA in buffering the detrimental effects of Cas9 expression and implicating secondary functions of Cas9 that warrant further studies. Our research defines a set of guidelines for generating Cas9-expressing cells with improved selection and transgene stability, which will aid future applications of CRISPR-Cas9 technologies in T. gondii and other species.

## RESULTS

### Improving the efficiency and stability of Cas9 expression.

**FIG 1:**
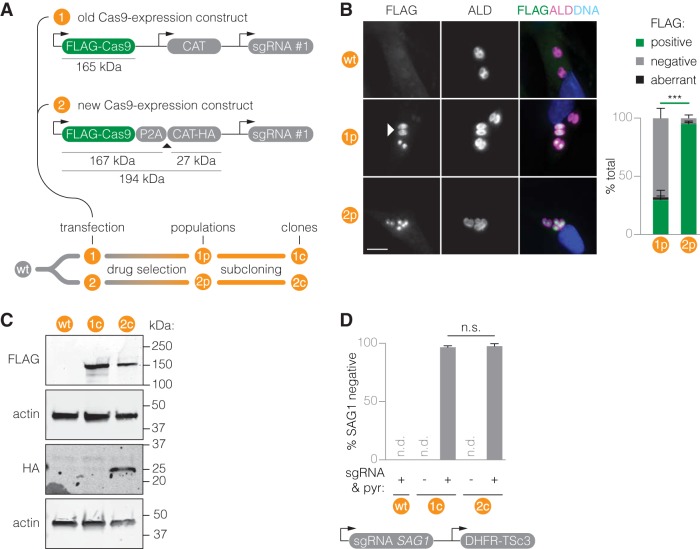
A construct for improved stability of Cas9 expression. (A) Schematic of Cas9 expression constructs. The triangle indicates the site of 2A-mediated peptide bond skipping. Expected molecular weights of processed and unprocessed proteins are indicated. Following transfection and chloramphenicol selection of these constructs in wt parasites, the resulting populations were subcloned. Populations after drug selection and clones were further assayed. (B) Representative immunofluorescence images of methanol-fixed intracellular parasites. Cells were stained for FLAG. ALD provides a cytosolic parasite stain, and Hoechst stain was used to stain host and parasite DNA. The white triangle marks a vacuole with aberrant, cytosolic FLAG localization. The bar graph shows the corresponding quantification of parasite vacuoles positive for FLAG staining. Bar, 10 μm. CAT, chloramphenicol acetyltransferase. ALD, aldolase. Mean ± SEM, *n *=* *3 biological replicates. Data were arcsine transformed prior to performing an unpaired *t* test. ***, *P ≤ *0.001. (C) Immunoblots showing processing of transcriptionally linked constructs into separate polypeptides in isolated Cas9^+^ clones. Actin serves as a loading control. (D) The efficiency of *SAG1* disruption in wt and isolated Cas9^+^ clones was measured via immunofluorescence microscopy following different treatments. Mean ± SEM, *n *=* *2 independent experiments; n.s., nonsignificant; n.d., not detected; pyr, pyrimethamine.

We previously generated Cas9-expressing parasites using constructs where both Cas9 and the drug resistance marker chloramphenicol acetyltransferase (CAT) are expressed from separate tubulin 1 (TUB1) promoters (23) (construct 1, Fig. 1A; see also Table S1 in the supplemental material). In this system, we have repeatedly observed spontaneous loss of Cas9 expression from clonal populations regardless of drug selection. To improve the stability of Cas9 expression, we therefore employed viral 2A “skip” peptides to closely couple Cas9 expression to drug resistance (construct 2). To test this 2A-linked expression system, and to compare it to our previous system, we separately transfected constructs 1 and 2 into the wild-type T. gondii strain RH (wt). Immunofluorescence staining and microscopy confirmed that nearly all vacuoles expressed the drug resistance marker (CAT^+^) in populations selected for construct 1 (1p) and populations selected for construct 2 (2p) (Fig. S1). About 95% of vacuoles in 2p expressed the nuclease (Cas9^+^), which was in stark contrast to the 30% Cas9^+^ vacuoles in 1p (Fig. 1B). We also found elevated levels of aberrant Cas9 localization to the cytosol. We conclude that the P2A-mediated transcriptional link enables a more stringent selection for Cas9 expression.

10.1128/mSphere.00386-19.1 FIG S1 Drug-selected parasites express CAT. Representative immunofluorescence images of methanol-fixed intracellular parasites. Cells were stained for CAT. ALD provides a cytosolic parasite stain, and Hoechst stain was used to stain host and parasite DNA. The bar graph shows the immunofluorescence assay-correspondent quantification of parasite vacuoles positive for CAT staining. Bar, 10 μm. CAT, chloramphenicol acetyltransferase; ALD, aldolase; n.s., nonsignificant. Mean ± SEM, *n *=* *3 biological replicates. Data were arcsine transformed prior to performing an unpaired *t* test. Download FIG S1, PDF file, 83.5 MB. Copyright © 2019 Markus et al. 2019 Markus et al. This content is distributed under the terms of the Creative Commons Attribution 4.0 International license.

10.1128/mSphere.00386-19.3 TABLE S1 Plasmids used in this study. Download Table S1, PDF file, 72.7 MB. Copyright © 2019 Markus et al. 2019 Markus et al. This content is distributed under the terms of the Creative Commons Attribution 4.0 International license.

To determine whether the P2A-linked Cas9-CAT transcript is indeed translated into discrete proteins, we derived clonal populations 1c and 2c from 1p and 2p, respectively. Immunoblotting of total parasite lysates resolved by SDS-PAGE demonstrated the presence of full-length FLAG-tagged Cas9 in both clonal populations and hemagglutinin (HA)-tagged CAT in population 2c (Fig. 1C). As with previously published uses of 2A peptides, some incomplete scission of the polypeptide could be observed in the 2c population (24).

We compared Cas9 function in the newly generated strains 1c and 2c by assessing gene disruption efficiency upon transfection and drug selection of a *SAG1*-targeting sgRNA. Immunofluorescence microscopy showed that the *SAG1* disruption efficiency was similar between strains 1c and 2c (Fig. 1D). In parallel efforts, we have successfully used strain 2c for CRISPR-mediated LOF screens and have not observed spontaneous loss of Cas9 expression since we switched to this new expression system (data not shown).

### A genome-targeting sgRNA increases the rate of successful Cas9 construct integration.

**FIG 2:**
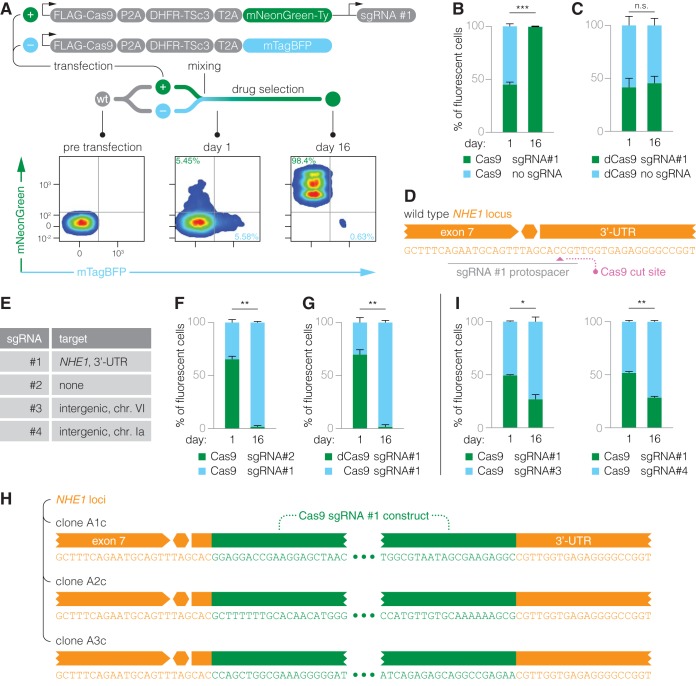
Genome targeting increases the rate of Cas9 construct integration. (A) Experimental outline of the fluorescence-based competition assays used in this study. Representative flow cytometry pseudocolor density plots are shown for parasites assessed at time points before, 1 day after, and 16 days after transfection of the constructs depicted above. (B) Bar graphs display the proportion of mNeonGreen- and mTagBFP-positive cells at day 1 posttransfection and mixing and day 16 following continuous drug selection. A Cas9 expression construct carrying sgRNA #1 outcompeted one lacking an sgRNA. (C) Results of an analogous competition in the presence or absence of sgRNA #1; here, selecting parasites for dCas9 expression. (D) sgRNA #1 is predicted to target Cas9 to the *NHE1* 3′ UTR in type I strains of T. gondii. The orange hexagon denotes the position of the stop codon. (E) Names and genomic targets of sgRNAs used in this study. (F) sgRNA #1 was highly favorable over an sgRNA with no genomic target (sgRNA #2) when selecting for Cas9 expression. (G) In the presence of sgRNA #1, a construct with Cas9 nuclease activity improved selection. (H) Whole-genome sequencing of clones derived from three independent transfections using a Cas9 construct expressing sgRNA #1 showed integration of the expression construct at the *NHE1* 3′ UTR. (I) Targeting alternative intergenic loci (sgRNAs #3 and #4) is comparable to targeting the *NHE1* locus via sgRNA #1. Mean ± SEM, *n *=* *2 biological replicates. Quantitative data were arcsine transformed prior to performing an unpaired *t* test. *, *P ≤ *0.05; **, *P ≤ *0.01; ***, *P ≤ *0.001; n.s., nonsignificant.

Initial failed attempts to generate stable Cas9-expressing parasites suggested an inherent toxicity of Cas9 in T. gondii. Only when we coexpressed an sgRNA, #1, were we able to isolate stable Cas9^+^ cells, which enabled the first genome-wide CRISPR-mediated LOF screens in apicomplexans (23). We therefore sought to exploit our new expression system to study the apparent buffering of Cas9 toxicity by the coexpressed sgRNA. To this end, we devised a fluorescence-based competition assay to assess relative parasite fitness in pairwise competitions. We generated an expression construct that encodes Cas9, a pyrimethamine-resistant allele of the bifunctional dihydrofolate reductase/thymidylate synthase (DHFR-TSc3) (25) as a selectable marker, and the fluorescent protein mNeonGreen as a single transcript, along with a U6-promoter-driven sgRNA #1 cassette (Fig. 2A). We used DHFR-TSc3 as the selectable marker over CAT to allow faster selection of resistant parasites. By immunoblotting, we confirmed that the tripartite transcript was translated into discrete proteins (Fig. S2A). We then derived an analogous construct devoid of an sgRNA expression cassette (sgRNA^–^) and carrying mTagBFP instead of mNeonGreen. Under selection for Cas9 expression, we wanted to test for differences in parasite fitness that could be associated with the presence or absence of sgRNA #1. To this end, we separately transfected wt parasites with the sgRNA^+^ and sgRNA^–^ constructs, mixed equal numbers of transfected cells, and cultured them with pyrimethamine for 16 days until parasites grew in a normal 2-day cycle. We determined the proportion of sgRNA^+^ (mNeonGreen^+^) to sgRNA^–^ (mTagBFP^+^) cells via flow cytometry upon first lysis and after drug selection. While the ratio was initially balanced, parasites carrying the sgRNA #1 construct readily outcompeted sgRNA^–^ parasites during the course of drug selection (Fig. 2B). This is the first formal evidence that sgRNA #1 provides a fitness advantage when selecting for Cas9 expression.

10.1128/mSphere.00386-19.2 FIG S2 Assessment of Cas9 fidelity in fitness and integration of various constructs. (A) Schematic of a representative Cas9 expression construct as was used in the fluorescence-based competition experiments. Triangles indicates sites of 2A-mediated peptide bond skipping. Expected molecular weights of processed and unprocessed proteins are indicated. Immunoblots show processing of transcriptionally linked constructs into separate polypeptides in isolated Cas9^+^ clones. Actin serves as a loading control. The triangle marks a FLAG^+^ protein band that corresponds to an unprocessed polypeptide. (B) Transfectants of Streptococcus pyogenes Cas9 variants with improved on-target specificity were outcompeted by wt Cas9, cotransfected with sgRNA #1. (C) An A-U-flipped and hairpin-extended sgRNA #1 scaffold conferred a marginal gain in parasite fitness against the unmodified sgRNA #1 scaffold. n.s., nonsignificant. Mean ± SEM, *n *=* *3 biological replicates. Data were arcsine transformed prior to performing an unpaired *t* test. ***, *P ≤ *0.001. (D) In the Cas9 strain that was generated with construct 2 from Fig. 1, parts of the Cas9 expression construct integrated directly downstream of the *NHE1* stop codon—at the site that is targeted by sgRNA #1. The modified locus was characterized by a set of PCRs for which the primer positions are shown. Primer sequences are provided in Table S4. Sanger sequencing traces for the 5′ and 3′ integration junctions are shown below. Download FIG S2, PDF file, 74.4 MB. Copyright © 2019 Markus et al. 2019 Markus et al. This content is distributed under the terms of the Creative Commons Attribution 4.0 International license.

To determine whether the effect of sgRNA #1 is linked to Cas9 nuclease activity, we generated corresponding constructs that encode catalytically dead Cas9 (dCas9). In competition, we observed no difference in fitness between dCas9 expression in the presence and in the absence of sgRNA #1, suggesting that the effect of the sgRNA is linked to Cas9 nuclease activity (Fig. 2C). Furthermore, we observed that increasing the specificity of Cas9 via previously published mutations did not obviate sgRNA #1 (Fig. S2B, Cas9-HF1 and eCas9) (26, 27). Conversely, we found that boosting sgRNA #1 transcription and binding to Cas9 by introducing a stem-loop A-U flip and hairpin extension (28) provided a marginal but additional gain in parasite fitness against the unmodified sgRNA #1 (Fig. S2C).

Initially, we believed sgRNA #1 to be nontargeting due to a mutation in the protospacer adjacent motif in type 2 strains of T. gondii. However, after publication of our previous study (23), we discovered that sgRNA #1 is indeed predicted to target the *NHE1* gene locus in type 1 strains within the 3′ untranslated region (UTR) (Fig. 2D). To determine the role of genome targeting as an sgRNA feature, we specifically designed a second sgRNA (sgRNA #2) to be truly nontargeting (Fig. 2E; Table S2). Parasites transfected with the targeting sgRNA #1 readily outcompeted parasites that received the nontargeting sgRNA #2, indicating that genome targeting is a favorable sgRNA feature in selecting for Cas9 expression (Fig. 2F).

10.1128/mSphere.00386-19.4 TABLE S2 sgRNA protospacers. Download Table S2, PDF file, 72.8 MB. Copyright © 2019 Markus et al. 2019 Markus et al. This content is distributed under the terms of the Creative Commons Attribution 4.0 International license.

To assess the role of nuclease activity directly, we also competed parasites transfected with one of two sgRNA #1 constructs: one expressing Cas9 and one expressing dCas9. We found that in the presence of sgRNA #1, Cas9 nuclease activity improved selection substantially (Fig. 2G). We concluded that introducing a DNA double-strand break (DSB) is the dominant effect behind sgRNA #1 improving Cas9 selection. For stable expression, the construct carrying the transgene must integrate into the genome of T. gondii since the parasite does not maintain episomal DNA (29). DSBs are thought to be the rate-limiting step in recombination, as these provide the required site for integrating constructs through NHEJ-mediated repair. For example, a previous study in T. gondii demonstrated that electroporation in the presence of restriction enzymes could increase construct integration efficiency 90-fold (30). We reasoned that Cas9-mediated DSBs may similarly increase the rate of construct integration. To determine whether the *NHE1* locus is ultimately targeted by sgRNA #1, we derived one clone each from three independent transfections of a Cas9 expression construct carrying sgRNA #1. We performed whole-genome sequencing on these clones and used an unbiased approach to identify construct integration sites by searching for reads that map chimerically to both the genome and the Cas9 construct. This analysis yielded a number of reads that mapped to the *NHE1* locus in all three clones (Table S3), enabling partial reconstruction of the edited *NHE1* loci (Fig. 2H). Single chimeric reads that mapped to other loci were deemed false positives based on reads confirming the integrity of those loci (Table S3). In the Cas9 strain (2c) generated with construct 2, we further confirmed integration at the *NHE1* locus by Sanger sequencing (Fig. S2D; Table S4). We also found that our previously generated Cas9 strain carries a large insertion at the *NHE1* locus. Based on its phenotype score in our genome-wide LOF screens in both strains, *NHE1* was not functionally disrupted by these integration events (23).

10.1128/mSphere.00386-19.5 TABLE S3 Chimerically mapping sequencing reads between sgRNA^+^ Cas9 expression construct and T. gondii genome (group A clones). Download Table S3, PDF file, 72.8 MB. Copyright © 2019 Markus et al. 2019 Markus et al. This content is distributed under the terms of the Creative Commons Attribution 4.0 International license.

10.1128/mSphere.00386-19.6 TABLE S4 Primers used to characterize the *NHE1* locus. Download Table S4, PDF file, 72.7 MB. Copyright © 2019 Markus et al. 2019 Markus et al. This content is distributed under the terms of the Creative Commons Attribution 4.0 International license.

To explore the possibility of targeting construct integration to other, potentially more favorable loci, we designed two sgRNAs (#3 and #4) to target neutral loci that were devoid of genes or known regulatory elements (chromosome [Chr.] VI, 1487284 to 1487304, and Chr. Ia, 1462133 to 1462153, in the T. gondii GT1 genome reference version 42). When selecting for Cas9 expression in competition with sgRNA #1, both of the new targeting guides (#3 and #4) conferred a minor fitness advantage (Fig. 2I), indicating that both neutral loci are appropriate for transgene integration.

### The sgRNA improves the fitness of Cas9-expressing parasites even after construct integration.

**FIG 3:**
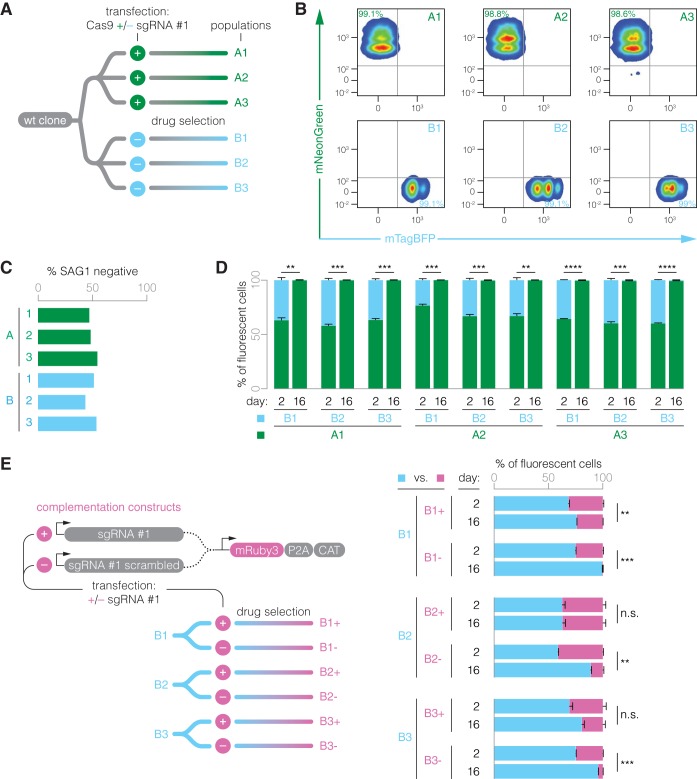
Long-term fitness cost of carrying Cas9 in the absence of an sgRNA. (A) Using improved constructs, three independent populations of Cas9-expressing parasites were generated for constructs carrying sgRNA #1 (group A) or lacking an sgRNA (group B). (B) Flow cytometry of drug-selected stable populations showed that parasites expressed the expected fluorescence marker. (C) Expression of functional Cas9 was confirmed by immunofluorescence microscopy, assessing the efficiency of *SAG1* gene disruption 24 h after transfection of a *SAG1*-targeting sgRNA. (D) All drug-selected populations generated in the presence of sgRNA #1 outcompeted those that lacked an sgRNA. (E) Group B populations were transfected with complementation constructs that encode the red fluorescent protein mRuby3 and sgRNA #1 or a nonfunctional, scrambled sequence. Drug-selected populations were competed against their parental population. For competition data, mean ± SEM, *n *=* *2 biological replicates. Data were arcsine transformed prior to performing an unpaired *t* test. **, *P ≤ *0.01; ***, *P ≤ *0.001; ****, *P ≤ *0.0001; n.s., nonsignificant.

We wondered whether the sgRNA may play an additional role in improving the fitness of Cas9-expressing parasites beyond mediating initial construct integration. We were able to generate sgRNA^–^/Cas9^+^ populations when selecting parasites in isolation, likely based on the stronger selection afforded by the 2A peptide design. If the sole advantage of sgRNA expression was to improve the integration of the Cas9 expression construct, we would not expect phenotypic differences between sgRNA^–^ and sgRNA^+^ populations following stable integration. To test for this hypothesis, we performed three independent transfections of the sgRNA^+^ and sgRNA^–^ constructs from Fig. 2A into a clonal wt population and drug selected the transfectants for Cas9 expression (Fig. 3A). Populations A1 to A3 were generated in the presence of sgRNA #1, and populations B1 to B3 were generated in the absence of an sgRNA. Following drug selection, we confirmed the expression of functional Cas9 in all populations by flow cytometry (Fig. 3B) and by testing the *SAG1* disruption efficiency following transfection of an *SAG1*-targeting sgRNA without selection (Fig. 3C). These populations were indistinguishable by these measures.

We then asked whether the drug-selected sgRNA^–^ populations retain a fitness defect compared to sgRNA^+^ populations. To test this, we performed nine pairwise competition assays between the independently derived populations. We mixed equal numbers of parasites and let them compete over the course of 16 days. Indeed, we observed that sgRNA^+^ populations consistently outcompeted sgRNA^–^ parasites, suggesting that Cas9 imparts a persistent fitness cost, which is alleviated by the presence of sgRNA #1 (Fig. 3D).

To unambiguously associate the continued fitness discrepancy with the presence or absence of the sgRNA, we designed an sgRNA #1-carrying complementation construct encoding an mRuby3 fluorescent marker and a chloramphenicol-selectable marker (Fig. 3E, left). Since transgenes often carry their own fitness cost, we also generated a control construct in which we randomized the sgRNA #1 sequence, including the sgRNA scaffold that mediates Cas9 binding. Group B populations were transfected with the complementation (B1^+^ to B3^+^) or control (B1^–^ to B3^–^) constructs. Following drug selection, these populations were competed against their parental populations (B1 to B3). We observed no difference or a slight fitness decrease in complemented parasites relative to their parental populations, consistent with a transgene-dependent fitness cost (Fig. 3E, right). Importantly, parasites that were selected for the control construct were significantly less fit than their parental populations—and, by extension, less fit than the complemented populations. Taken together, these results indicate that sgRNA expression counteracts Cas9 toxicity and thereby improves long-term fitness.

### The fitness cost of Cas9 expression persists in clonal populations.

**FIG 4:**
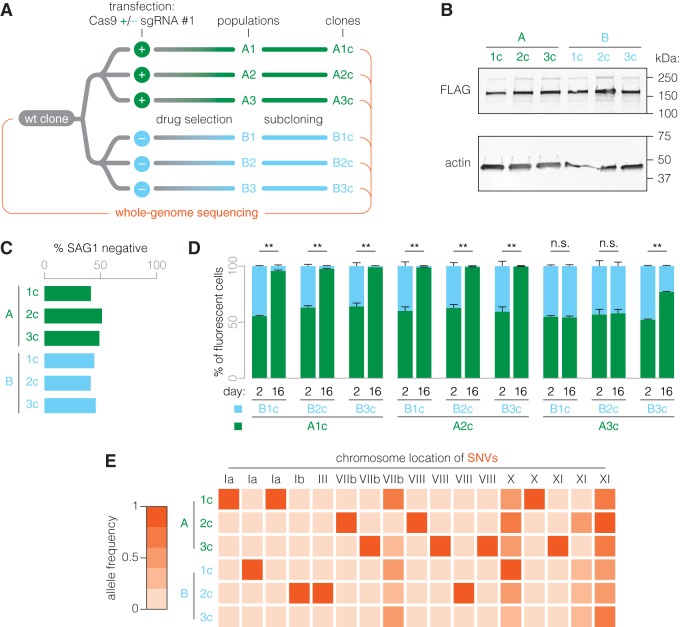
The fitness cost of Cas9 expression in the absence of an sgRNA persists in clonal populations. (A) Clonal populations were derived from populations of groups A and B. (B) Immunoblot showing Cas9 expression among clones A1c to A3c and B1c to B3c. Cas9 expression was detected by blotting for FLAG. Actin serves as a loading control. (C) Expression of functional Cas9 was confirmed by immunofluorescence microscopy, assessing the efficiency of *SAG1* gene disruption 24 h after transfection of a *SAG1*-targeting sgRNA. (D) Clonal populations largely recapitulated the results of their parental populations, in that group A clones generally outcompeted clones of group B. Only clone A3c was equally as fit as or less fit than group B clones. Mean ± SEM, *n *=* *2 biological replicates. Data were arcsine transformed prior to performing an unpaired *t* test. **, *P ≤ *0.01; n.s., nonsignificant. (E) Whole-genome sequencing was performed on group A and B clones and the parental wt clone. Numbers of SNVs relative to the parental wt clone occurred at a normal rate and were similar between clones of groups A and B. No SNVs were group specific.

To assess whether the fitness defect in Cas9^+^ populations lacking an sgRNA is recapitulated at a clonal level, we derived one clone from each of the stable drug-selected populations: A1c to A3c and B1c to B3c (Fig. 4A). Immunoblotting confirmed similar levels of full-length Cas9 expression across all clones (Fig. 4B). As with construct 2, we observed a minor portion of uncleaved transgene. *SAG1* disruption efficiencies, as assessed upon transfection but without selection of a *SAG1*-targeting sgRNA, were similar across all clones, confirming the presence of functional Cas9 (Fig. 4C). Analogously to the population-level competitions, we performed nine pairwise competitions between clones of group A and clones of group B and saw that the fitness defect in sgRNA^–^ populations was recapitulated in their clonal derivatives (Fig. 4D). However, we found that the sgRNA^+^ clone A3c was similar to or only slightly fitter than the group B clones. In light of the population results, A3c likely represents an outlier.

We hypothesized that Cas9 may exhibit directed or undirected nuclease activity against the genome of T. gondii and that sgRNA #1 may alleviate toxicity by modulating Cas9 activity. Consequently, we aimed to find genomic signatures that would be indicative of Cas9 nuclease activity or mechanisms of adaptation to Cas9 expression in the absence of sgRNA #1. The group A and group B clones and their parental wt strain provided the clean genetic background needed for this analysis. We performed whole-genome sequencing on these seven clonal populations and aligned the data with the T. gondii GT1 reference genome. As in group A clones (Fig. 2H; Table S3), we found singular construct integration sites for group B clones (Table S5). However, while group A clones all showed construct integration at the site of sgRNA #1 targeting adjacent to the *NHE1* locus, the integration sites in group B clones mapped to different loci in chromosomes VIIa (B1c), XII (B2c), and IX (B3c). Furthermore, we found no copy number variations or structural (long) insertions or deletions that were not already present in the parental strain. We identified single-nucleotide variants (SNVs) that had arisen in clonal populations since diverging from our parental wt (Table S6). Excluding SNVs originating from erroneous read mapping or other technical causes, we found that none of the valid SNVs were group specific and that clones from the two groups had acquired similar numbers of SNVs (Fig. 4E).

10.1128/mSphere.00386-19.7 TABLE S5 Chimerically mapping sequencing reads between sgRNA^–^ Cas9 expression construct and T. gondii genome (group B clones). Download Table S5, PDF file, 72.8 MB. Copyright © 2019 Markus et al. 2019 Markus et al. This content is distributed under the terms of the Creative Commons Attribution 4.0 International license.

10.1128/mSphere.00386-19.8 TABLE S6 SNVs identified in clones A1c to A3c and B1c to B3c. Download Table S6, PDF file, 72.8 MB. Copyright © 2019 Markus et al. 2019 Markus et al. This content is distributed under the terms of the Creative Commons Attribution 4.0 International license.

Taken together, we found that the fitness cost imposed by Cas9 expression and its relief by the presence of sgRNA #1 persist in clonal populations. However, we found no genomic signatures that would support a mechanism of Cas9 toxicity mediated by targeted or nonspecific nuclease activity. Cas9 has been shown to undergo extensive structural rearrangements upon sgRNA binding (10, 14). Consequently, sgRNA #1 may modulate Cas9 toxicity by stabilizing a less-toxic protein fold.

## DISCUSSION

Our initial studies suggested that Cas9 expression in the absence of an sgRNA is toxic for T. gondii. Spontaneous loss of Cas9 from strains and the subsequent expansion of the Cas9^–^ population have been common problems in genome-wide screens. Here, we present a Cas9 expression system that improves selection and transgene stability. Characterizing the role of the coexpressed sgRNA, we found that its primary function is to enhance construct integration by targeting Cas9 to create a DNA DSB. However, we determined that the coexpressed sgRNA also improves parasite fitness even after construct integration. We were unable to find signatures indicative of targeted or nonspecific nuclease activity when analyzing the genomes of Cas9-expressing strains derived in the presence or absence of the sgRNA; nevertheless, in view of the evidence, we suspect that toxicity arises from nonspecific nuclease activity in the absence of an sgRNA. Collectively, our work develops guidelines that will aid the generation of Cas9-expressing strains in diverse organisms, mitigating collateral effects from the nuclease.

Using viral 2A peptides to directly couple the expression of Cas9 and a drug resistance marker greatly increased the efficiency of selection and the stability of Cas9-expressing strains. Constitutive expression of Cas9 was initially achieved using constructs that expressed Cas9 and the drug resistance marker from separate promoters (23), which resulted in frequent spontaneous loss of Cas9 expression, despite continuous drug selection. Viral 2A peptides transcriptionally link Cas9 expression to drug selection, making loss of transgene expression less likely. Indeed, our lab has not observed loss of Cas9 expression in these strains. A recent study similarly demonstrated that 2A peptides can stabilize the expression of dimerizable-Cre-recombinase (DiCre) subunits to generate a highly efficient inducible knockout strain (31). We also observed that initial generation of transgenic populations was more effective with the 2A peptide, increasing the proportion of selected parasites expressing the transgene from 30% to 95%. Similar selection strategies are important in a variety of genetic approaches and will enable future functional analyses of transgenes in T. gondii.

Stable transgene expression relies upon construct integration into the genome, since T. gondii does not maintain episomal DNA (29). Early studies in T. gondii and mammalian cells demonstrated that DSBs, induced following electroporation in the presence of restriction enzymes, substantially increased construct integration efficiencies (30, 32). Consequently, we found that sgRNAs targeting any of three different loci improved selection for Cas9 expression, by targeting construct integration to the cleavage site. However, sgRNAs did not improve selection for dCas9. In this context, selection for Cas9 expression is highly favored over dCas9 expression, despite the expected fitness cost associated with DSBs. Cas9 activity can therefore be exploited to target construct integration to discrete loci in T. gondii and other organisms that exhibit highly active NHEJ pathways.

The fitness of Cas9-expressing parasites continues to be enhanced by the coexpressed sgRNA long after successful integration. The enhanced selection efficiency of the 2A peptide expression system enabled us to select for stable Cas9 expression even in the absence of a targeting sgRNA. Compared to sgRNA^+^ cells, these sgRNA^–^ cells retained a fitness defect. Complementing the expression of sgRNA #1 in sgRNA^–^ cells, we found that the presence or absence of the sgRNA likely accounts for the observed fitness discrepancy. The persisting fitness defect in Cas9-expressing sgRNA^–^ cells and the continued efficacy of the sgRNA #1 in alleviating this phenotype suggest that Cas9 expression impairs parasite fitness and that the sgRNA modulates this effect.

We found no evidence for increased nonspecific nuclease activity in the absence of the sgRNA, as the overall mutation rate was low across all clones, and no other genomic variations were detected. We also found no evidence for directed nuclease activity or a mechanism of adaptation to Cas9 expression since SNVs were mostly unique to specific clones and occurred in isolation, rather than clustered. To the best of our knowledge, the observed fitness difference between populations and clones has to be attributed to the presence or absence of the sgRNA, which may prevent nonspecific activity from Cas9. If such nonspecific activity largely results in parasite death, signatures of the increased nuclease activity might not have accumulated over the relatively short propagation period of the sequenced clones.

The sgRNA #1 may modulate Cas9 toxicity by stabilizing a less-toxic protein fold. Cas9 toxicity has been observed in many prokaryotes (10–12) as well as other eukaryotes (13–15). Overexpression of transgenes can impair cellular fitness in multiple ways. It is conceivable that both Cas9 and dCas9 interfere with cellular homeostasis via their DNA- and RNA-binding abilities. Indeed, Cas9 and dCas9 have been shown to bind PAM sequences nonspecifically, especially in the absence of an sgRNA, which may contribute to the observed toxicity (20–22). Consequently, the sgRNA #1 may stabilize a Cas9 fold that is more compatible with cell viability. In T. gondii, however, we specifically found that nuclease activity underlies Cas9 toxicity by observing that the sgRNA #1 improved parasite fitness only in the presence of Cas9 but was irrelevant for dCas9 constructs. Similar observations were reported in the bacterium Corynebacterium glutamicum, where viable dCas9^+^ strains were generated, but repeated attempts to express Cas9 failed (12). Structural studies have shown that, following sgRNA binding, Cas9 undergoes large conformational changes essential for cleavage of target DNA (33, 34). However, while these conformational changes are required for sgRNA-dependent nuclease activity, DNA and Mn^2+^ have previously been shown to trigger conformational changes that enable sgRNA-independent DNA cleavage by Cas9 (35). The mechanism by which sgRNA #1 promotes parasite fitness in the presence of Cas9 therefore appears 2-fold: by facilitating construct integration following initial genome targeting and by subsequently acting as a nontargeting “decoy” sgRNA to block sgRNA-independent activity. We conclude that, while we lack a definitive understanding of its mode of action, the coexpressed sgRNA improves parasite fitness in the presence of Cas9 and should be included in future strain generations.

Our observations suggest that coexpressing an sgRNA may stabilize Cas9 expression and protect the cell from Cas9 toxicity in other systems. With its ease, versatility, and broad applicability, the CRISPR-Cas9 system is used to probe biological processes in a continuously growing number of species. Specifically, major efforts are being undertaken toward developing CRISPR-Cas9–based genome editing for therapeutic applications in humans (reviewed in reference 36). Here, Cas9 off-target activity has been understood as a major hurdle to its clinical use, which has motivated work to increase on-target specificity (reviewed in reference 37). Typically, the off-target activity of Cas9 is evaluated and considered solely in the context of sgRNAs. Our work indicates that Cas9 expression in the absence of an sgRNA is toxic in T. gondii, and we hypothesize that this toxicity is mediated by a secondary nontargeted nuclease activity. Coexpressing sufficient levels of sgRNA to completely sequester all Cas9 molecules present at any given time may safeguard the genome from aberrant nuclease activity, which has implications for Cas9 as a tool for both biological studies and therapeutic applications.

## MATERIALS AND METHODS

### Parasites and host cells.

T. gondii tachyzoites from strain RH and derived strains were maintained at 37°C with 5% CO_2_ growing in human foreskin fibroblasts (HFFs; ATCC SCRC-1041) cultured in Dulbecco’s modified Eagle’s medium (DMEM) supplemented with 3% or 10% heat-inactivated fetal bovine serum and 10 μg/ml gentamicin. Transgenic parasites were obtained by electroporation of constructs into cells and selection with 40 μM chloramphenicol (Sigma-Aldrich) or 3 μM pyrimethamine (Sigma-Aldrich). Clones were isolated by limiting dilution.

### Transfection.

Freshly egressed parasites were passed through a polycarbonate filter with a 3-μm pore size (Whatman) and then washed and resuspended in Cytomix (38) (10 mM KPO_4_, 120 mM KCl, 150 mM CaCl_2_, 5 mM MgCl_2_, 25 mM HEPES, 2 mM EDTA) to 8 × 10^7^ cells/ml. Two hundred forty-five microliters of the parasite suspension was combined with 50 μg of previously linearized plasmid in 155 μl Cytomix, supplemented with 2 mM ATP, 5 mM glutathione (GSH), to a final volume of 400 μl. Parasites were electroporated in 4-mm-gap cuvettes (BTX Harvard Apparatus model no. 640) in an Electro Square Porator (BTX Harvard Apparatus) set to 1.7 kV, for two 176-μs pulses at 100-ms intervals.

### Immunoblotting.

Freshly egressed parasites were filtered and washed in PBS before lysis in 2× Laemmli buffer (4% SDS, 20% glycerol, 5% 2-mercaptoethanol, 0.02% bromophenol blue, 120 mM Tris-HCl, pH 6.8). Samples were heated to 100°C for 5 min prior to resolution by SDS-PAGE. After transferring the separated proteins onto nitrocellulose at a constant 25 V overnight, membranes were blocked for 1 h at room temperature in TBS-T, 5% (wt/vol) nonfat dry milk. Immunoblots were probed, as indicated, with rabbit antialdolase (anti-ALD) (WU1614 [39]) diluted 1:2,000, mouse anti-α-tubulin (12G10; Developmental Studies Hybridoma Bank) diluted 1:10,000, mouse anti-FLAG (M2; Sigma-Aldrich) diluted 1:5,000, mouse anti-CAT (MAB3678; EMD Millipore monoclonal) diluted 1:1,000, rabbit anti-actin (TgACT1) (40) diluted 1:10,000, mouse anti-Ty (BB2 [41]) diluted 1:5,000, mouse anti-2A peptide (3H4; Novus Biologicals) diluted 1:1,000, or mouse anti-HA (16B12; BioLegend) diluted 1:2,000. The signal was detected using 1:20,000 dilutions of IRDye 800CW-conjugated goat anti-mouse IgG and IRDye 680CW-conjugated donkey anti-rabbit IgG (Li-Cor Biosciences) on an Odyssey infrared imager (Li-Cor Biosciences).

### Immunofluorescence staining and microscopy.

Intracellular parasites were fixed on glass coverslips at 4°C with either methanol for 2 min or 4% formaldehyde for 10 min. Methanol fixation was used when probing for FLAG-Cas9 and CAT. Formaldehyde fixation was used when probing for SAG1. Formaldehyde-fixed samples were permeabilized with 0.25% Triton X-100 in PBS for 8 min. Mouse monoclonal antibodies were used to detect SAG1 (clone DG52 [42], FLAG-tagged Cas9 [clone M2; Sigma-Aldrich], and CAT [MAB3678; EMD Millipore monoclonal]). Rabbit polyclonal serum was used to detect ALD (WU1614 [39]). Primary antibodies were detected with Alexa Fluor-labeled secondary antibodies. Nuclei were stained with Hoechst stain (Santa Cruz), and coverslips were mounted in Prolong Diamond (Thermo Fisher). Images were acquired using an Eclipse Ti epifluorescence microscope (Nikon) using the NIS elements imaging software. Adobe Photoshop was used for image processing. Per technical replicate, 100 parasite vacuoles were identified based on aldolase staining, prior to determining the number of vacuoles positive for SAG1, FLAG, or CAT.

### Flow cytometry.

Freshly egressed parasites were filtered, pelleted at 1,000 × *g*, and resuspended in PBS before adding an equal volume of 8% formaldehyde in PBS. Following incubation for 15 min at room temperature, parasites were pelleted and washed twice before resuspending in PBS. Fluorescence from mNeonGreen, mTagBFP, and mRuby3 was detected on a FACSCanto II (BD Biosciences). Flow cytometry data were acquired with FACSDiva software (BD Biosciences) and analyzed using FlowJo.

### Fluorescence-based competition assay.

For competitions of transfected parasites, plasmids were linearized by restriction-enzyme digest with PvuI (New England BioLabs [NEB]). Fifty micrograms of the respective linearized construct was then transfected into 2 × 10^7^ parasites. Equal numbers of transfected cells (10^7^ cells each) were mixed for pairwise competitions. The day after transfection, the overinfected HFF monolayers lysed, and egressed parasites were analyzed by flow cytometry to determine the initial ratio of mNeonGreen^+^ to mTagBFP^+^ cells. Starting at 24 h posttransfection, parasites were maintained in the presence of 3 μM pyrimethamine to select for construct integration and Cas9 expression. Competing populations typically had resumed a normal 2-day growth cycle by day 16 posttransfection, when the final fluorescence ratio was determined. Parasites were passed between host cell monolayers soon after natural egress, and in high inocula, to mitigate population drifts due to bottlenecks. Each competition entailed two biological replicates from two independently transfected populations per construct. Two technical replicates were maintained per biological replicate. For competitions of stable drug-selected and clonal populations, equal numbers of freshly egressed parasites were mixed. Parasites were maintained in the presence of 3 μM pyrimethamine. For the sgRNA #1 complementation experiments, chloramphenicol was washed out from stable populations, prior to mixing with their chloramphenicol-sensitive parental populations. Upon first monolayer lysis, 2 days postmixing, the initial fluorescence ratio (mTagBFP to mNeonGreen or mRuby3) was determined by flow cytometry. Mixed populations were propagated for an additional 14 days before determining the final fluorescence ratio by flow cytometry.

### Determining efficiency of *SAG1* disruption.

pU6[sgSAG1]-DHFR (Addgene no. 80322) was linearized by restriction digest with AseI (NEB) and dialyzed against distilled water (dH_2_O). Parasites were transfected with 50 μg of the linearized plasmid and seeded on HFFs at a multiplicity of infection (MOI) of 10. Pyrimethamine was added 24 h posttransfection. Upon egress from host cells 2 days after transfection, parasites were used to infect HFFs seeded on coverslips. SAG1 loss was quantified by immunofluorescence 24 h after infecting coverslips, which was equivalent to 3 days posttransfection. For populations in which the Cas9 expression construct was selected for with pyrimethamine, parasites were transfected with 50 μg of undigested pU6[sgSAG1]-DHFR, followed by infecting HFFs seeded on coverslips and quantifying SAG1 loss by immunofluorescence at 24 h posttransfection.

### Statistics.

SAG1, FLAG, and CAT immunofluorescence data as well as mNeonGreen, mTagBFP, and mRuby3 flow cytometry data were arcsine transformed prior to conducting unpaired two-tailed Student's *t* tests.

### Genome sequencing.

Freshly egressed parasites were filtered and washed in PBS before DNA extraction using the DNeasy Blood & Tissue kit (Qiagen). Next-generation sequencing libraries were prepared using the TruSeq DNA PCR-free kit (Illumina). Sequencing was conducted using 300-nt paired-end reads on an Illumina NextSeq instrument, resulting in an average 22-fold coverage. Reads were aligned using Burrows-Wheeler Aligner (“bwa mem”) (43) to the T. gondii GT1 reference genome from ToxoDB (version 39) plus the respective integrant sequence, using default parameters. SNVs were identified with “bcftools mpileup” v1.9 (with option –B) (44) and filtered with vcf-annotate (v0.1.14, with default filters). Copy number analysis was performed by analyzing read depth with CNVnator (45) using a bin size of 1,000. To determine the construct integration sites in clones of groups A and B, we appended an artificial contig to the T. gondii GT1 genome assembly that contained the sequence of the sgRNA^+^ (group A) or the sgRNA^–^ (group B) construct. We then used STAR (46) to align quality-trimmed sequencing reads separately as single-end reads to the corresponding genome, disabling spliced alignments but enabling chimeric alignments with a minimum 20-nt read length on each side of the chimeric junction. Reads that were chimeric with the expression construct were extracted and are listed in Tables S2 and S4 in the supplemental material.

### Plasmids.

Table S1 details all plasmids and their use within this study. The corresponding plasmid maps can be found in the GenBank database via the indicated accession numbers. The plasmid for generating stable Cas9-expressing T. gondii (pBM019, also referred to as construct 2 in this study) is available from Addgene. The remaining plasmids are available upon request.

### Data availability.

Plasmid maps have been deposited in GenBank under accession numbers MN019110 to MN019124.
