# Supplementary figures and images for: Optimizing Systems for Cas9 Expression in Toxoplasma gondii
Source: mSphere. 2019 Jun 26;4(3):e00386-19. doi: 10.1128/mSphere.00386-19 (PMC6595152; doi:10.1128/mSphere.00386-19)

Figure S1

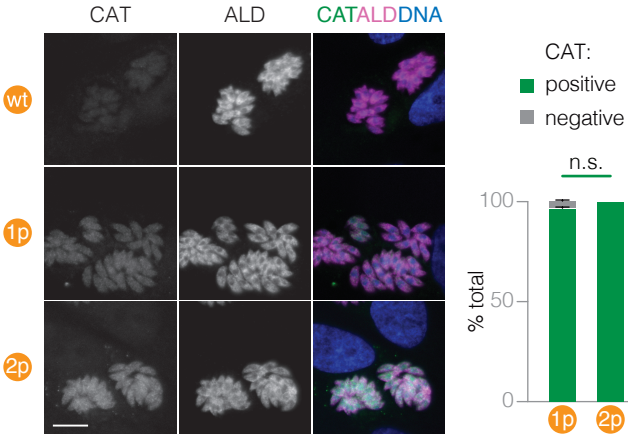

Supplement: FIG S1 [file mSphere.00386-19-sf001.pdf]

Figure S2

A

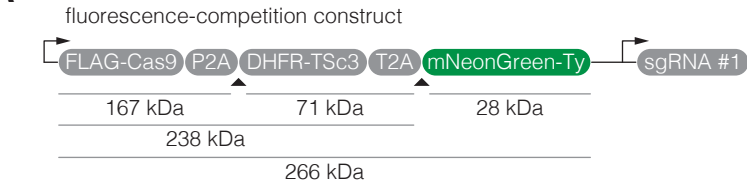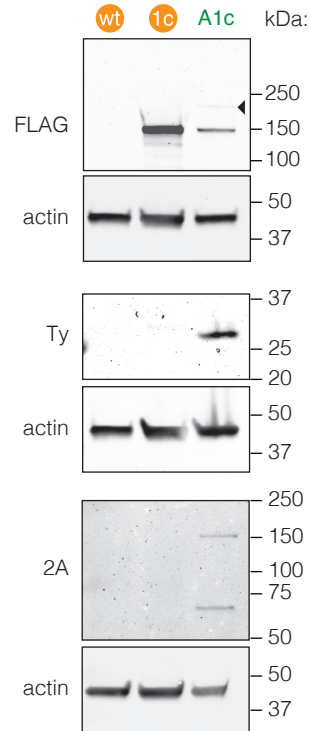

B

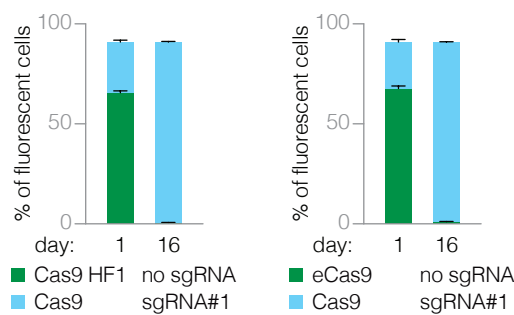

C

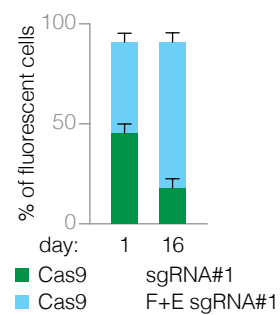

D

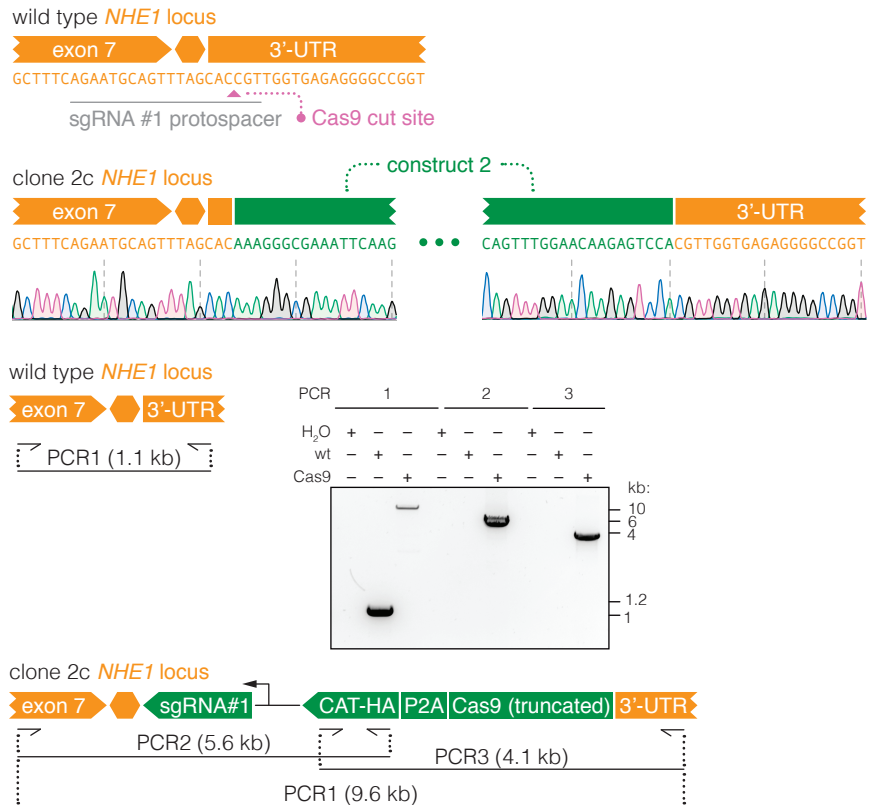

Supplement: FIG S2 [file mSphere.00386-19-sf002.pdf]
