# Supplementary material for: Optimizing Systems for Cas9 Expression in Toxoplasma gondii
Source: mSphere. 2019 Jun 26;4(3):e00386-19. doi: 10.1128/mSphere.00386-19 (PMC6595152; doi:10.1128/mSphere.00386-19)
Supplement: TABLE S1 [file mSphere.00386-19-st001.pdf]

Table S1: Plasmids used in this study

plasmid name	description	used in figures	Addgene	GenBank
pBM062	used to generate Cas9-expressing cells (construct 1)	1; S1	–	MN019114
pBM019	used to generate Cas9-expressing cells (construct 2)	1; S1; S2B	#128179	MN019123
pU6[sgSAG1]-DHFR	*SAG1*-targeting sgRNA (Sidik et al., 2014 [5])	1D, 3C, 4C	#80322	–
pBM006	sgRNA #1 and Cas9; used for competitions	2A,B,I,H; 3A–D; 4A–E; S2A,D	–	MN019111
pBM013	Cas9; used for competitions	2A,B; 3A–E; 4A–E	–	MN019124
pBM020	sgRNA #1 and dCas9; used for competitions	2C,G	–	MN019122
pBM023	dCas9; used for competitions	2C	–	MN019121
pBM040	sgRNA #2 and Cas9; used for competitions	2F	–	MN019117
pBM025	sgRNA #1 and Cas9; used for competitions	2F,G; S2C	–	MN019120
pBM041	sgRNA #3 and Cas9; used for competitions	2I	–	MN019116
pBM042	sgRNA #4 and Cas9; used for competitions	2I	–	MN019115
pBM064	mRuby3 and sgRNA #1 (complementation construct); used for competitions	3E	–	MN019113
pBM065	mRuby3 and a scrambled version of sgRNA #1 (complementation-control construct); used for competitions	3E	–	MN019112
pBM027	sgRNA #1 and eCas9	S2C	–	MN019110
pBM029	sgRNA #1 and Cas9-HF1	S2C	–	MN019118
pBM026	flip-extended sgRNA #1 (F+E sgRNA#1) and Cas9	S2D	–	MN019119
