# Supplementary material for: Optimizing Systems for Cas9 Expression in Toxoplasma gondii
Source: mSphere. 2019 Jun 26;4(3):e00386-19. doi: 10.1128/mSphere.00386-19 (PMC6595152; doi:10.1128/mSphere.00386-19)
Supplement: TABLE S2 [file mSphere.00386-19-st002.pdf]

Table S2: sgRNA protospacers

sgRNA	target	protospacer sequence
#1	*NHE1* 3'-UTR	GAGAATGCAGTTTAGCACCGT
#2	no predicted target in *T. gondii* GT1	GTGAGTTAGCGTCGAACTCG
#3	intergenic region (chr. VI: 1,487,284–1,487,304)	GCCGTTCTGTCTCACGATGC
#4	intergenic region (chr. Ia: 1,462,133–1,462,153)	GATCGAGTCAGCTCAGATAT
