# Supplementary material for: Optimizing Systems for Cas9 Expression in Toxoplasma gondii
Source: mSphere. 2019 Jun 26;4(3):e00386-19. doi: 10.1128/mSphere.00386-19 (PMC6595152; doi:10.1128/mSphere.00386-19)
Supplement: TABLE S3 [file mSphere.00386-19-st003.pdf]

**Table S3:** Chimerically-mapping sequencing reads between sgRNA$^{+}$ Cas9-expression construct and *T. gondii* genome (group A clones)

| | contig A | contig A coordinate | contig A strand | contig B | contig B coordinate | contig B strand | CIGAR (contig A) | CIGAR (contig B) |
|---|---|---|---|---|---|---|---|---|
| clone A1c | AAQM03000805 | 5576 | - | pBM006 | 10623 | - | 70S147M | 70M147S |
| | pBM006 | 1762 | - | TGGT1_chrVIIb | 3940177 | + | 73S130M | 130S73M |
| | pBM006 | 3617 | - | TGGT1_chrXII | 2563189 | - | 86S212M | 86M212S |
| | pBM006 | 526 | - | TGGT1_chrVIIb | 2842781 | + | 189S92M19S | 111S189M |
| | pBM006 | 526 | - | TGGT1_chrVIIb | 2842781 | + | 123S92M | 92S123M |
| | pBM006 | 526 | - | TGGT1_chrVIIb | 2842781 | + | 127S88M | 88S127M |
| | pBM006 | 526 | - | TGGT1_chrVIIb | 2842781 | + | 170S83M | 83S170M |
| | pBM006 | 8059 | - | AAQM03000001 | 145 | - | 108S193M | 108M193S |
| | TGGT1_chrIb | 950047 | + | pBM006 | 4444 | - | 104M193S | 193M104S |
| | TGGT1_chrIV | 2264912 | - | pBM006 | 1964 | - | 103S197M | 103M197S |
| | TGGT1_chrVIIb | 2842782 | - | pBM006 | 525 | + | 30S178M | 178S30M |
| | TGGT1_chrVIIb | 2842782 | - | pBM006 | 525 | + | 71S145M | 145S71M |
| | TGGT1_chrVIIb | 2842782 | - | pBM006 | 525 | + | 45S189M | 189S45M |
| | TGGT1_chrX | 583086 | + | pBM006 | 6495 | - | 185M103S | 103M185S |
| clone A2c | pBM006 | 11208 | + | TGGT1_chrVIIb | 2842782 | + | 177M124S | 177S124M |
| | pBM006 | 6818 | + | TGGT1_chrXI | 3149611 | + | 248M48S | 248S48M |
| | pBM006 | 11209 | + | TGGT1_chrVIIb | 2842783 | - | 206M25S | 25M206S |
| | pBM006 | 11209 | + | TGGT1_chrVIIb | 2842783 | - | 31M182S | 182M31S |
| | pBM006 | 11208 | + | TGGT1_chrVIIb | 2842782 | + | 45M149S | 45S149M |
| | pBM006 | 11208 | + | TGGT1_chrVIIb | 2842782 | + | 160M55S | 160S55M |
| | pBM006 | 11208 | + | TGGT1_chrVIIb | 2842782 | + | 238M56S | 238S56M |
| | pBM006 | 11208 | + | TGGT1_chrVIIb | 2842782 | + | 246M55S | 246S55M |
| | TGGT1_chrVIIb | 2842783 | + | pBM006 | 11209 | - | 43M257S | 257M43S |
| | pBM006 | 11208 | + | TGGT1_chrVIIb | 2842782 | + | 266M35S | 266S35M |
| | pBM006 | 11209 | + | TGGT1_chrVIIb | 2842783 | - | 232M65S | 65M232S |
| | pBM006 | 4892 | - | TGGT1_chrX | 4509421 | - | 149S150M | 149M150S |
| | pBM006 | 11208 | + | TGGT1_chrVIIb | 2842782 | + | 103M134S | 103S134M |
| | pBM006 | 11208 | + | TGGT1_chrVIIb | 2842782 | + | 72M187S | 72S187M |
| | TGGT1_chrX | 574673 | + | pBM006 | 10134 | + | 108M80S | 108S80M |
| | pBM006 | 11208 | + | TGGT1_chrVIIb | 2842782 | + | 140M156S | 140S156M |
| | pBM006 | 7789 | + | TGGT1_chrXII | 4295071 | - | 146M148S | 148M146S |
| | pBM006 | 11209 | + | TGGT1_chrVIIb | 2842783 | - | 161M111S | 111M161S |
| | TGGT1_chrVIIb | 2842783 | + | pBM006 | 11209 | - | 174M126S | 126M174S |
| | TGGT1_chrVIIb | 2842783 | - | pBM006 | 11209 | - | 114S98M | 114M98S |
| | TGGT1_chrVIIb | 2842783 | + | pBM006 | 11209 | - | 36M118S | 118M36S |
| | TGGT1_chrVIIb | 2842783 | + | pBM006 | 11209 | - | 145M154S | 154M145S |
| | pBM006 | 11208 | + | TGGT1_chrVIIb | 2842782 | + | 258M43S | 258S43M |
| | pBM006 | 11209 | + | TGGT1_chrVIIb | 2842783 | - | 105M117S | 117M105S |
| | pBM006 | 7247 | + | TGGT1_chrIa | 1490829 | - | 176M118S | 118M176S |
| | pBM006 | 11208 | + | TGGT1_chrVIIb | 2842782 | + | 64M220S | 64S220M |
| | pBM006 | 11209 | + | TGGT1_chrVIIb | 2842783 | - | 218M83S | 83M218S |
| clone A3c | AAQM03000805 | 5576 | - | pBM006 | 10623 | - | 70S147M | 70M147S |
| | pBM006 | 526 | - | TGGT1_chrVIIb | 2842781 | + | 189S92M19S | 111S189M |
| | pBM006 | 4374 | - | TGGT1_chrVIIb | 2580925 | + | 204S95M | 95S204M |
| | pBM006 | 526 | - | TGGT1_chrVIIb | 2842781 | + | 179S29M | 29S179M |
| | TGGT1_chrVIIb | 2842782 | - | pBM006 | 525 | + | 107S192M | 192S93M14S |
| | pBM006 | 526 | - | TGGT1_chrVIIb | 2842781 | + | 123S92M | 92S123M |
| | pBM006 | 5370 | + | AAQM03000741 | 5460 | - | 221M72S | 72M221S |
| | pBM006 | 526 | - | TGGT1_chrVIIb | 2842781 | + | 127S88M | 88S127M |
| | pBM006 | 10622 | + | AAQM03000805 | 5575 | + | 168M131S | 168S131M |
| | TGGT1_chrX | 583086 | + | pBM006 | 6495 | - | 185M103S | 103M185S |
| | pBM006 | 8059 | - | AAQM03000001 | 145 | - | 108S193M | 108M193S |
| | pBM006 | 1762 | - | TGGT1_chrVIIb | 3940177 | + | 73S130M | 130S73M |
| | pBM006 | 526 | - | TGGT1_chrVIIb | 2842781 | + | 146S70M | 70S146M |
| | pBM006 | 3617 | - | TGGT1_chrXII | 2563189 | - | 86S212M | 86M212S |
| | pBM006 | 526 | - | TGGT1_chrVIIb | 2842781 | + | 170S83M | 83S170M |
| | TGGT1_chrVI | 580409 | + | pBM006 | 6125 | + | 194M106S | 194S106M |
| | pBM006 | 526 | - | TGGT1_chrVIIb | 2842781 | + | 190S92M5S | 97S190M |
| | pBM006 | 526 | - | TGGT1_chrVIIb | 2842781 | + | 239S62M | 62S239M |
| | TGGT1_chrVIIb | 2842782 | - | pBM006 | 525 | + | 108S191M | 191S93M15S |
| | pBM006 | 180 | - | TGGT1_chrVIII | 3081782 | + | 56S224M | 224S56M |
| | pBM006 | 1964 | + | TGGT1_chrIV | 2264912 | + | 271M30S | 271S30M |
| | pBM006 | 4444 | + | TGGT1_chrIb | 950047 | - | 205M95S | 95M205S |
| | pBM006 | 526 | - | TGGT1_chrVIIb | 2842781 | + | 222S77M | 77S222M |
