# Supplementary material for: Optimizing Systems for Cas9 Expression in Toxoplasma gondii
Source: mSphere. 2019 Jun 26;4(3):e00386-19. doi: 10.1128/mSphere.00386-19 (PMC6595152; doi:10.1128/mSphere.00386-19)
Supplement: TABLE S4 [file mSphere.00386-19-st004.pdf]

Table S4: Primers used to characterize the *NHE1* locus

PCR	forward primer	reverse primer
PCR1	AGTTCCGTCTGTCACGGTTC	CGCTGGAACAACATGACGC
PCR2	AGTTCCGTCTGTCACGGTTC	GTGTTCACCCTTGTTACACCG
PCR3	CGTAACACGCCACATCTTGC	CGCTGGAACAACATGACGC
