# Supplementary material for: Optimizing Systems for Cas9 Expression in Toxoplasma gondii
Source: mSphere. 2019 Jun 26;4(3):e00386-19. doi: 10.1128/mSphere.00386-19 (PMC6595152; doi:10.1128/mSphere.00386-19)
Supplement: TABLE S5 [file mSphere.00386-19-st005.pdf]

Table S5: Chimerically-mapping sequencing reads between sgRNA^{-} Cas9-expression construct and *T. gondii* genome (group B clones)

	contig A	contig A coordinate	contig A strand	contig B	contig B coordinate	contig B strand	CIGAR (contig A)	CIGAR (contig B)
clone B1c	pBM013	3410	-	TGGT1_chrVIIa	2995119	-	105S116M	105M116S
	pBM013	3407	-	TGGT1_chrVIIa	2995116	-	131S139M	131M139S
	pBM013	3407	-	TGGT1_chrVIIa	2995116	-	118S174M	118M174S
	pBM013	3407	-	TGGT1_chrVIIa	2995116	-	89S118M	89M118S
	pBM013	3407	-	TGGT1_chrVIIa	2995116	-	173S77M	173M77S
	pBM013	8662	+	AAQM03000886	5610	-	150M143S	143M150S
	pBM013	1271	-	TGGT1_chrVIIa	2995119	+	254S32M6S	38S254M
	pBM013	3407	-	TGGT1_chrVIIa	2995116	-	55S245M	55M245S
	pBM013	3407	-	TGGT1_chrVIIa	2995116	-	209S91M	209M91S
	pBM013	3407	-	TGGT1_chrVIIa	2995116	-	88S178M	88M178S
	pBM013	3407	-	TGGT1_chrVIIa	2995116	-	114S168M	114M168S
	pBM013	1271	-	TGGT1_chrVIIa	2995119	+	157S32M4S	36S157M
	TGGT1_chrVIIa	2995113	+	pBM013	3404	+	121M138S	121S138M
	TGGT1_chrVIIa	2995113	+	pBM013	3404	+	94M190S	94S190M
	TGGT1_chrVIIa	2995113	+	pBM013	3404	+	91M208S	91S208M
	TGGT1_chrVIIa	2995113	+	pBM013	3404	+	30M210S	30S210M
	TGGT1_chrVIIa	2995113	+	pBM013	3404	+	87M213S	87S213M
	TGGT1_chrVIIa	2995119	-	pBM013	1271	+	45S183M	183S32M13S
	TGGT1_chrVIIa	2995113	+	pBM013	3404	+	81M138S	81S138M
	TGGT1_chrVIIa	2995113	+	pBM013	3404	+	99M200S	99S200M
	TGGT1_chrXI	4762075	-	pBM013	7334	+	74S227M	227S74M
clone B2c	pBM013	1473	-	TGGT1_chrVIIa	598605	+	53S228M	228S53M
	pBM013	10564	+	TGGT1_chrXII	364945	-	180M70S	70M180S
	pBM013	502	-	TGGT1_chrXII	364944	+	175S116M10S	126S175M
	pBM013	502	-	TGGT1_chrXII	364944	+	134S66M	66S134M
	pBM013	502	-	TGGT1_chrXII	364944	+	165S79M	79S165M
	pBM013	502	-	TGGT1_chrXII	364944	+	231S42M	42S231M
	pBM013	10564	+	TGGT1_chrXII	364945	-	103M161S	161M103S
	pBM013	502	-	TGGT1_chrXII	364944	+	198S98M	98S198M
	pBM013	502	-	TGGT1_chrXII	364944	+	269S32M	32S269M
	pBM013	10564	+	TGGT1_chrXII	364945	-	109M76S	76M109S
	pBM013	502	-	TGGT1_chrXII	364944	+	202S71M	71S202M
	pBM013	502	-	TGGT1_chrXII	364944	+	209S85M	85S209M
	pBM013	10564	+	TGGT1_chrXII	364945	-	172M128S	128M172S
	pBM013	10564	+	TGGT1_chrXII	364945	-	83M211S	211M83S
	pBM013	502	-	TGGT1_chrXII	364944	+	28S114M	114S28M
	pBM013	7739	+	TGGT1_chrIV	916000	-	232M68S	68M232S
	pBM013	10564	+	TGGT1_chrXII	364945	-	130M169S	169M130S
	pBM013	10564	+	TGGT1_chrXII	364945	-	147M80S	80M147S
	pBM013	502	-	TGGT1_chrXII	364944	+	200S85M	85S200M
	pBM013	502	-	TGGT1_chrXII	364944	+	213S37M	37S213M
	pBM013	10564	+	TGGT1_chrXII	364945	-	65M153S	153M65S
	pBM013	9800	-	TGGT1_chrXI	1919806	+	174S126M	126S174M
	pBM013	3359	+	AAQM03000794	8539	+	198M102S	198S102M
	pBM013	10564	+	TGGT1_chrXII	364945	-	171M89S	89M171S
	pBM013	1723	+	TGGT1_chrIX	2587497	-	172M127S	127M172S
	pBM013	10564	+	TGGT1_chrXII	364945	-	81M121S	121M81S
	TGGT1_chrII	497829	+	pBM013	7616	+	214M87S	214S87M
	TGGT1_chrXII	364945	-	pBM013	501	+	74S106M	106S59M15S
	TGGT1_chrXII	364940	+	pBM013	10569	-	37M257S	257M37S
	TGGT1_chrXII	364945	-	pBM013	501	+	74S201M	201S74M
clone B3c	pBM013	4307	+	TGGT1_chrX	2720203	+	116M170S	116S170M
	pBM013	486	+	TGGT1_chrIX	3830920	-	191M80S	80M191S
	pBM013	483	+	TGGT1_chrIX	3830923	-	159M86S	86M159S
	pBM013	10593	-	TGGT1_chrIX	3830919	+	73S228M	228S73M
	pBM013	483	+	TGGT1_chrIX	3830923	-	149M97S	97M149S
	pBM013	483	+	TGGT1_chrIX	3830923	-	56M240S	240M56S
	pBM013	10593	-	TGGT1_chrIX	3830919	+	75S224M	224S75M
	pBM013	483	+	TGGT1_chrIX	3830923	-	30M189S	189M30S
	pBM013	514	-	TGGT1_chrX	2720201	-	197S52M	197M52S
	pBM013	10593	-	TGGT1_chrIX	3830919	+	32S222M	222S32M
	pBM013	5245	+	TGGT1_chrVIIb	4563458	-	52M223S	223M52S
	pBM013	3677	+	TGGT1_chrVIII	5730254	-	60M240S	240M60S
	pBM013	483	+	TGGT1_chrIX	3830923	-	186M115S	115M186S
	pBM013	10593	-	TGGT1_chrIX	3830919	+	75S191M	191S75M
	pBM013	10593	-	TGGT1_chrIX	3830919	+	151S121M	121S151M
	pBM013	10593	-	TGGT1_chrIX	3830919	+	90S160M	160S89M1S
	pBM013	10593	-	TGGT1_chrIX	3830919	+	207S59M	59S207M
	pBM013	483	+	TGGT1_chrIX	3830923	-	75M225S	225M75S
	pBM013	10593	-	TGGT1_chrIX	3830919	+	185S47M	47S185M
	pBM013	483	+	TGGT1_chrIX	3830923	-	160M139S	139M160S
	pBM013	10593	-	TGGT1_chrIX	3830919	+	82S218M	218S82M
	pBM013	10827	-	TGGT1_chrX	4575439	+	136S165M	165S136M
	pBM013	483	+	TGGT1_chrIX	3830923	-	212M79S	79M212S
	pBM013	483	+	TGGT1_chrIX	3830923	-	115M108S	108M115S
	pBM013	10593	-	TGGT1_chrIX	3830919	+	269S26M	26S269M
	pBM013	10080	+	TGGT1_chrVI	2411728	+	163M127S	163S127M
	pBM013	483	+	TGGT1_chrIX	3830923	-	128M111S	111M128S
	pBM013	10593	-	TGGT1_chrIX	3830919	+	86S199M	199S86M
	pBM013	10593	-	TGGT1_chrIX	3830919	+	44S177M	177S44M
	pBM013	483	+	TGGT1_chrIX	3830923	-	66M218S	218M66S
	pBM013	483	+	TGGT1_chrIX	3830923	-	76M224S	20S204M76S
	pBM013	10593	-	TGGT1_chrIX	3830919	+	104S92M	92S104M
	TGGT1_chrIX	3830923	+	pBM013	483	-	68M219S	219M68S
	TGGT1_chrIX	3830919	-	pBM013	10593	+	190S88M	88S190M
	TGGT1_chrIX	3830923	+	pBM013	483	-	56M245S	245M56S
	TGGT1_chrIX	3830923	+	pBM013	483	-	60M239S	239M60S
