# Supplementary material for: Optimizing Systems for Cas9 Expression in Toxoplasma gondii
Source: mSphere. 2019 Jun 26;4(3):e00386-19. doi: 10.1128/mSphere.00386-19 (PMC6595152; doi:10.1128/mSphere.00386-19)
Supplement: TABLE S6 [file mSphere.00386-19-st006.pdf]

Table S6: SNVs identified in clones A1c–A3c and B1c–B3c

assembly	chromosome	coordinate	alleles	annotation	comments	alternative allele frequencies A1c	A2c	A3c	B1c	B2c	B3c
TGGT1_v.39	chrIa	1381322	C/G	TGGT1_295090	Pro > Arg	1.000	0.000	0.000	0.000	0.000	0.000
TGGT1_v.39	chrIa	1798184	G/C	TGGT1_295340	Glu > Asp	0.000	0.000	0.000	0.950	0.000	0.000
TGGT1_v.39	chrIa	482224	A/G	TGGT1_293660	Trp > Arg	0.950	0.000	0.000	0.000	0.000	0.000
TGGT1_v.39	chrIb	126440	T/G	TGGT1_207480	intronic	0.000	0.000	0.000	0.000	1.000	0.000
TGGT1_v.39	chrIII	1742320	G/A	TGGT1_254470	intronic	0.000	0.000	0.000	0.000	1.000	0.000
TGGT1_v.39	chrIII	981220	C/T	intergenic	read derived from expression construct	0.608	0.636	0.630	0.000	0.000	0.000
TGGT1_v.39	chrIII	981292	C/A	intergenic	read derived from expression construct	0.500	0.636	0.632	0.023	0.000	0.000
TGGT1_v.39	chrVIIb	2347359	T/C	TGGT1_260310	intronic	0.000	1.000	0.000	0.000	0.000	0.000
TGGT1_v.39	chrVIIb	2426986	C/T	TGGT1_260210	intronic	0.000	0.000	1.000	0.000	0.000	0.000
TGGT1_v.39	chrVIIb	3745777	A/G	intergenic		0.674	0.148	0.394	0.442	0.373	0.425
TGGT1_v.39	chrVIII	3902027	C/A	intergenic		0.000	1.000	0.000	0.000	0.000	0.000
TGGT1_v.39	chrVIII	4989894	C/A	intergenic		0.000	0.033	1.000	0.000	0.000	0.000
TGGT1_v.39	chrVIII	6718028	C/G	intergenic		0.000	0.000	0.000	0.000	1.000	0.000
TGGT1_v.39	chrVIII	910523	T/C	intergenic		0.000	0.000	0.962	0.000	0.000	0.000
TGGT1_v.39	chrX	4530963	T/A	intergenic		0.532	0.686	0.515	0.962	0.579	0.368
TGGT1_v.39	chrX	5487482	G/C	TGGT1_236930	Ser > Trp	1.000	0.000	0.000	0.000	0.000	0.000
TGGT1_v.39	chrXI	2421184	A/G	intergenic		0.000	0.000	1.000	0.000	0.000	0.000
TGGT1_v.39	chrXI	26152	T/C	intergenic		0.033	0.462	0.179	0.271	0.306	0.318
TGGT1_v.39	chrXI	5681307	C/T	TGGT1_216970	read derived from apicoplast genome	0.455	0.000	0.241	0.034	0.292	0.083
TGGT1_v.39	chrXI	5681333	C/T	TGGT1_216970	read derived from apicoplast genome	0.481	0.000	0.300	0.065	0.400	0.120
TGGT1_v.39	chrXI	5681337	G/A	TGGT1_216970	read derived from apicoplast genome	0.500	0.000	0.286	0.065	0.400	0.111
TGGT1_v.39	chrXI	5681341	C/A	TGGT1_216970	read derived from apicoplast genome	0.500	0.000	0.308	0.065	0.400	0.111
TGGT1_v.39	chrXI	5681345	C/A	TGGT1_216970	read derived from apicoplast genome	0.500	0.000	0.300	0.067	0.423	0.111
TGGT1_v.39	chrXI	5681348	G/A	TGGT1_216970	read derived from apicoplast genome	0.500	0.000	0.300	0.067	0.385	0.111
TGGT1_v.39	chrXI	5681360	C/T	TGGT1_216970	read derived from apicoplast genome	0.520	0.000	0.286	0.067	0.345	0.103
TGGT1_v.39	chrXI	5681367	C/T	TGGT1_216970	read derived from apicoplast genome	0.520	0.000	0.310	0.065	0.310	0.107
TGGT1_v.39	chrXI	5681368	C/T	TGGT1_216970	read derived from apicoplast genome	0.520	0.000	0.310	0.065	0.310	0.111
TGGT1_v.39	chrXI	5681378	G/A	TGGT1_216970	read derived from apicoplast genome	0.520	0.000	0.293	0.063	0.286	0.125
TGGT1_v.39	chrXI	7948	T/A	intergenic		0.662	0.964	0.606	0.596	0.500	0.623

Gray SNVs were called on the basis of erroneous read mapping.
